# Adverse maternal environment and western diet impairs cognitive function and alters hippocampal glucocorticoid receptor promoter methylation in male mice

**DOI:** 10.14814/phy2.14407

**Published:** 2020-04-25

**Authors:** Xingrao Ke, Qi Fu, Jennifer Sterrett, Cecilia J. Hillard, Robert H. Lane, Amber Majnik

**Affiliations:** ^1^ Division of Neonatology Department of Pediatrics Medical College of Wisconsin Milwaukee WI USA; ^2^ Neuroscience Research Center Rodent Behavior Core Department of Pharmacology and Toxicology Medical College of Wisconsin Milwaukee WI USA; ^3^ Children’s Mercy Research Institute Kansas City MO USA

**Keywords:** adverse maternal environment, DNA methylation, GR, hippocampus, western diet

## Abstract

Adverse maternal environment (AME) and high‐fat diet in early childhood increase the risk of cognitive impairment and depression later in life. Cognitive impairment associates with hippocampal dysfunction. A key regulator of hippocampal function is the glucocorticoid receptor. Increased hippocampal GR expression associates with cognitive impairment and depression. Transcriptional control of GR relies in part upon the DNA methylation status at multiple alternative initiation sites that are tissue specific, with exon 1.7 being hippocampal specific. Increased exon 1.7 expression associates with upregulated hippocampal GR expression in early life stress animal models. However, the effects of AME combined with postweaning western diet (WD) on offspring behaviors and the expression of GR exon 1 variants in the hippocampus are unknown. We hypothesized that AME and postweaning WD would impair cognitive function and cause depression‐like behavior in offspring in conjunction with dysregulated hippocampal expression of total GR and exon 1.7 variant in mice. We found that AME‐WD impaired learning and memory in male adult offspring concurrently with increased hippocampal expression of total GR and GR 1.7. We also found that increased GR 1.7 expression was associated with decreased DNA methylation at the GR 1.7 promoter. We speculate that decreased DNA methylation at the GR 1.7 promoter plays a role in AME‐WD induced increase of GR in the hippocampus. This increased GR expression may subsequently contribute to hippocampus dysfunction and lead to the cognitive impairment seen in this model.

## Graphical abstract

The central findings of this study are that AME and WD together decrease learning and memory in male adult offspring. This impaired learning and memory was concurrent with changes in hippocampal GR1.7 expression, DNA methylation, and GR signaling relative to AME or postweaning WD alone. This combination is relevant considering the previous literature associating neurocognitive impairment and hippocampal GR biology.

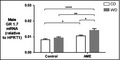

## 1 INTRODUCTION

An adverse maternal environment (AME) and the consumption of a western diet (WD) starting in early childhood affect health later in life (Alastalo et al., 2013; Francis & Stevenson, 2013). More specifically, human and animal studies reveal a strong association between exposure to either an AME or WD and the development of cognitive impairment and depressive disorders later in life (Alastalo et al., 2013; Arcego et al., 2016; Francis et al., 2013). AME has been shown to decreased learning and memory while maternal WD consumption has been shown to alter memory and depression in offspring (Alastalo et al., 2013; Arcego et al., 2016; de Kloet, Joels, & Holsboer, 2005; Francis et al., 2013). However, while AME and WD are often studied separately, these two conditions are often experienced together and over the course of a lifetime. We therefore developed a mouse model of chronic adversity to determine whether postweaning WD consumption exacerbated the impact of AME on cognitive function and a potential mechanism.

Exposure to stress and adversity as well as a western diet both alter glucocorticoid receptor levels in the hippocampus (Ke et al., 2010; Khazen, Hatoum, Ferreira, & Maroun, 2019; Szymanska et al., 2009). Interestingly, increased hippocampal glucocorticoid levels are associated with impaired cognitive function (Arp et al., 2016; Pillai et al., 2018). This is due in‐part to the regulation of hippocampal function through hippocampal glucocorticoid receptor (Zhang et al., 2019). GR is a transcription factor and a master regulator of hippocampal development and the stress response (Meijer, Buurstede, & Schaaf, 2019). GR activity is modulated in part by phosphorylation (Chen et al., 2008; Wang, Frederick, & Garabedian, 2002) and cochaperone proteins including the FK506‐binding protein 52 (FKBP4) and FK506‐binding protein 51 (FKBP5) (Storer, Dickey, Galigniana, Rein, & Cox, 2011). Phosphorylation of GR at Serine 211 (pGRser211) promotes GR nuclear translocation and enhances GR transcriptional activity (Chen et al., 2008). pGRser211 can serve as a biomarker for activated GR in vivo (Wang et al., 2002). FKBP4 also promotes GR translocation into the nuclear compartment and activates GR activity (Davies, Ning, & Sanchez, 2002). Once in the nucleus, GR initiates transcription of target genes including FKPB5 (Binder, 2009). Importantly, FKPB5 functions as a feedback molecule by binding to GR and subsequently inhibiting it, creating an important regulatory pathway for GR in the hippocampus. Interestingly, disrupting the interaction between GR, FKBP4, and FKBP5 is associated with cognitive impairment and depressive disorders (Fujii et al., 2014; Simic et al., 2013; Tatro, Everall, Kaul, & Achim, 2009).

Regulation of GR activity depends in part upon GR transcription levels. Transcriptional regulation of the GR gene relies in part on the regulation of multiple alternative initiation sites within exon 1 (McCormick et al., 2000; Turner & Muller, 2005). The GR gene is highly conserved between species. The mouse GR gene promoter is closely related to that of rat and human (Bockmuhl et al., 2011). Alternative splicing of mouse GR exon 1 generates 12 mRNA variants that are tissue specific (Bockmuhl et al., 2011; McCormick et al., 2000; Turner et al., 2005). Of which exon 1.7 in rodents is equivalent to exon 1F in humans and is expressed broadly in mouse (Bockmuhl et al., 2011) and in human (McGowan et al., 2009; Presul, Schmidt, Kofler, & Helmberg, 2007; Turner et al., 2005) brain tissue, respectively. Dysregulated exon 1.7/1F expression in the hippocampus is associated with early life events in both rodents and humans (Ke et al., 2010; McGowan et al., 2009; Weaver et al., 2004).

Transcriptional regulation of GR exon 1 variants also involves epigenetic mechanisms, including DNA methylation. When located in a gene promoter, reduced DNA methylation typically correlates with enhanced gene expression (Zemach, McDaniel, Silva, & Zilberman, 2010). DNA hypomethylation of the GR 1.7/1F promoter is associated with the increased expression of exon 1.7 and total GR in adult hippocampus exposed to maternal separation or maternal behavior in rodents and childhood abuse in humans (McGowan et al., 2009; Weaver et al., 2004). These observations suggest that the usage of this promoter could be altered by an adverse environment (Weaver et al., 2004). Unfortunately, the effects of the combination of an AME and postweaning WD on adult GR 1.7 variant expression and DNA methylation at GR 1.7 promoter have not been studied. Understanding the effects of AME and WD on GR may provide a potential marker and target for the effect of an early life adverse environment.

We therefore sought to determine how an AME and postweaning WD would affect behavior and GR transcriptional regulation. We hypothesized that the combination of AME and postweaning WD would impair cognition and induce depression‐like behavioral changes as well as dysregulate hippocampal expression of total GR and GR 1.7 variant in mice. We further hypothesized that dysregulated hippocampal GR 1.7 expression would be associated with altered DNA methylation status at its promoter.

## 2 METHODS

### 2.1 Animals

**Figure 1:**
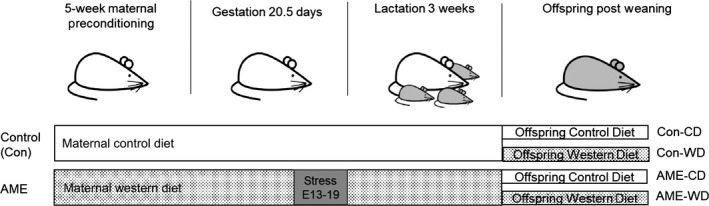
A diagram of adverse maternal environment and diet. Six‐week‐old female mice were subject to either a control diet (CD) or a Western diet (WD) for 5 weeks prior to pregnancy and throughout lactation. Environmental stressors were introduced on embryonic (E) days 13–19. At postnatal week 3, pups from both control (Con) and adverse maternal environment (AME) groups were weaned and permanently placed on either a CD or WD, creating four experimental groups: Con‐CD, Con‐WD, AME‐CD, and AME‐WD

All experiments were conducted according to the Public Health Services Policy on Human Care and Use of Laboratory Animals and all procedures were approved by the Medical College of Wisconsin Institutional Animal Care and Use Committee (American Physiological et al., 2002). The mouse model of AME used in this study was generated as follows: 6‐week‐old C57/Bl6 female mice were randomly subject to either a control diet (3.9 kcal/gram, 10% fat without cholesterol and sucrose, Research Diet Inc., New Brunswick, NJ, Product# D14020502) or a Western diet (4.67 kcal/gram, 40% fat, comprised of increased saturated fat, cholesterol, and sucrose, Research Diet Inc., Product# D12079B) for 5 weeks prior to pregnancy and throughout lactation. Dams in the control diet group experienced a normal environment throughout pregnancy and are designated as Control (Con). Dams fed a Western diet experienced a “stressed” environment the last third of pregnancy. The combination of chronic western diet and gestational stress is designated as adverse maternal environment (AME). The stressed environment consisted of daily random environmental changes as well as a static change in the maternal environment consisting of one of three of the standard amount of bedding from embryonic day (E)13 to E19. The acute random environmental changes included altered light cycles on 3 nonconsecutive days, three repeat cages throughout the day on E15, and the short‐term introduction of a novel object in the cage for a day. The period of prenatal stress was limited to E13‐E19 to minimize newborn mortality, avoid interference with implantation and still target a period of rapid development and environmental vulnerability. Dams’ body weights (BW) were recorded after 5 weeks exposed to CD or WD. Dams exposed to WD were significantly heavier than those exposed to CD by 13.3% (22.82 ± 1.24 gram and 20.13 ± 1.03 gram, respectively, ****p < .0001). Increased BW of WD dams were less than average +3 standard deviations (*SD*) of CD dams (23.22 gram), indicating WD dams were overweight compared to CD dams (Enriori et al., 2007). Dams delivered spontaneously, and litters were culled to six. No difference in litter size was noted between Con and AME. At postnatal week 3, pups from both control and AME groups were weaned and permanently placed on either a control diet (CD) or Western diet (WD), creating four experimental groups: Con‐CD, Con‐WD, AME‐CD, AME‐WD (Figure 1). Three to five pups of the same sex were housed per cage. Body weights (BW) and food intake were recorded weekly. Food intake recorded ended at week 11 of life. Calorie consumption was calculated by multiplying the energy content (kcal/gram) of each food by the amount of food consumed at the end of the week per mouse per day. Equal food intake by all mice in the cage was assumed. All behavior and molecular studies were carried out in adult animals starting at 12 weeks of age. Brains were quickly removed and weighed. Hippocampi (HP) were dissected and flash frozen in liquid nitrogen and stored in −80°C for future analysis. A total of 40 pregnant female mice were used in the study with *N* = 8 dams per group.

### 2.2 Behavioral tests

All behavior included a number of eight mice per sex per group from different litters. Mice underwent behavior tests in the order indicated below. Nonoverlapping groups of mice were used for the sucrose preference test and forced swim test.

1. *Open Field Test (OFT)* Mice were observed in an open field arena as a measure of anxiety‐like behavior and general locomotion activity. The open field arena used in this study was 120 × 120 × 30 cm. The arena was painted white and divided into 16 equal quadrants (30 × 30 cm) by black lines. For testing, mice were placed in the central quadrant and left to explore for 5 min. Mice were monitored and recorded by an overhead camera (Hitachi 2500A) approximately 2 m above the open field box. A 10% acetic acid solution was used to clean the apparatus between tests. The mean speed of travel, total distance traveled, mean number of center zone entries, and distance traveled in center zone were measured using Stoelting ANY‐maze software (Wood Dale, IL).
2. *Elevated Plus Maze *Mice were subjected to elevated plus maze test as a measure of anxiety‐like behavior. The elevated‐plus maze (San Diego Instruments, San Diego, CA) consisted of two open arms (30 × 5 cm) and two enclosed arms (30 × 5 × 15 cm) that extended from a central platform (5 × 5 cm). The maze was elevated 40 cm above the floor. Experiments began by placing a single mouse on the central platform facing an open arm. During the first 5 min of free exploration, the number of entries into (defined as an animal placing all four paws onto an arm) and time spent in open and closed arms were recorded by a trained observer blind to treatment condition. Percent of open arm entries, percent of time in open arm, total distance travelled in open arm and times of center entries were measured Stoelting ANY‐maze software (Wood Dale, IL).
3. *Morris Water Maze (MWM) *Learning and memory was assessed by a 6‐day version of the Morris Water Maze test consisting of 1 day of visible platform training followed by 4 days of hidden platform training and 1 day of spatial reversal trials. Briefly, a white circular polypropylene pool (100 cm in diameter and 20 cm in height) was filled with opaque water (made opaque with nonfat milk protein). On the pool rim, four points were designated (north, east, south, and west), dividing the pool into four quadrants (NE, NW, SW, and SE). On the first day of training, a platform was 8 × 8 cm positioned at the center of the NW quadrant, with standing area 1 cm above water's surface. The mouse was positioned in the water from random start points and ended when the mouse reached and escaped onto the platform. If unable to find the platform in 60 s, the mouse was gently guided to the platform. Mice were allowed to remain on the platform for 15 s. Mice were given four trials per day every 5 min. On the next 4 days, a hidden platform was submerged 1 cm below the water's surface at the center of the NW quadrant. On the last day (probe trial), the hidden platform was moved to the center of SE quadrant and mice were given four trials. The time taken to reach the platform was recorded for each trial. Swimming motivation, ability, or speed were also noted for each trial.
4. *Sucrose Preference Test (SPT) *Mice were habituated to the presence of two drinking bottles for at least 3 days in their home cages prior to testing. After the acclimatization the mice are separated into individual cages and presented with two drinking bottles: one containing 2% sucrose and the other water for 3 days in their home cages. Water and sucrose solution intake were measured daily by weighing the bottles. The positions of two bottles were switched daily to reduce any confound produced by a side bias. Sucrose preference is calculated as a percentage of the volume of sucrose intake over the total volume of fluid intake and averaged over the 3 days of testing.
5. *Forced Swim Test (FST) *Mice subjected to forced swimming test as a measure of depression‐like behavior. Two‐liter glass beakers (14.5 cm diameter and 18.5 cm height) were filled to a depth of 12 cm, which did not allow the mice to touch the bottom of the beaker with their hindlimbs when their heads were above water. The water temperature was 24 ± 2°C. Swim behavior was recorded and analyzed by scorers blinded to the experimental group. Time immobile was defined as no movement other than that required to maintain the animal's balance or keep its head above water. Immobility was scored during the last 4 min of total 6 min swim test.

### 2.3 Serum Corticosterone ELISA

Serum was collected from all four experimental groups. Briefly, whole blood was collected at the same time of day from each animal immediately following their death. Serum was collected by centrifuging whole blood 700 *g* at 4°C for 10 min. Serum corticosterone levels were measured using a corticosterone enzyme immunoassay kit (Cat# K014‐H1, Arbor Assays) following the manufacturer's instructions.

### 2.4 RNA Isolation and Real‐Time RT‐PCR

Total hippocampal RNA extraction and cDNA syntheses were performed as previously described (Cohen et al., 2016). mRNA levels of GR (Mm.PT.58.42952901**,** Integrated DNA Technologies**),** FKBP4 **(**Mm.PT.58.5267114, Integrated DNA Technologies)**,** FKBP5 (Mm.PT.58.10937155, Integrated DNA Technologies), and GR 1.7 mRNA variants were calculated relative to hypoxanthine phosphoribosyltransferase 1 (HPRT1, Mm.PT.39a.22214828, Integrated DNA Technologies) which was used as an internal control. Primer and probe sequences for GR 1.7 variantare listed in Table 1.

**Table 1:** Primers

Primers and probe for GR 1.7 variant real time RT‐PCR	
Forward	5’CCT CCC AGG CCA GTT AAT ATTT
Reverse	5’TATACAAGTCCATCACGCTTCC
Probe	5’TGGACTCCAAAGAATCCTTAGCTCCC
Primers for GR 1.7 pyrosequencing	
Forward	5’ GGTTTTGTAGGTTGGTTGTTATT
Reverse	5’ CTTTAATTTCTCTTCTCCCTAACTC
Sequence	5’ ATTTTTTAGGGGGTTTTGG

### 2.5 DNA Isolation and Pyrosequencing

Hippocampal DNA was extracted using Purelink genomic DNA mini kit (Thermo Fisher Scientific, Cat#K1820‐01). DNA was subjected to sodium bisulfite treatment using Epitech fast DNA bisulfite kit (Qiagen, Cat#59824) as per the manufacturer's protocol to determine site specific CpG methylation. DNA methylation of the validation‐set samples was determined through PCR amplification with biotinylated primers (Intergrated DNA Technologies, Coralville, IA). Primers were designed using PyroMark Assay Design Software version 2.0. Amplified products were confirmed with agarose gel electrophoresis. Percent of methylation was quantified by PyroMark Q48 Autoprep pyrosequencer (Qiagen, Valencia, CA). The primers used to examine DNA CpG methylation status in GR I.7 promoter are listed in Table 1.

### 2.6 Protein Isolation and Immunoblot

Hippocampal tissue proteins isolation and immunoblots were performed as previously described (Cohen et al., 2016). Antibodies against GR (Santa Cruz Biotechnology Inc, Cat# sc‐8992), phospho‐GR Ser 211 (Cell Signaling, Cat# 4161), FKBP4 (Cell Signaling, Cat# 11826S) and FKBP5 (Santa Cruz Biotechnology Inc., Cat#sc‐13983) and at 1:50 dilution were used to determine protein abundance and Vinculin (Cell Signaling, Cat #13901) at 1:10,000 dilution was used as a loading control.

### 2.7 Statistics

GraphPad Prism 6 (GraphPad Software, San Diego, CA) was used to perform all analyses. All data presented are expressed as mean ± *SEM*. Four‐group comparisons were analyzed by two‐way ANOVA followed by post‐hoc Tukey's multiple comparisons test. Two‐group comparisons were analyzed by Mann–Whitney (nonparametric) test. Significance was set as *p* < .05.

## 3 RESULTS

### 3.1 AME‐WD increased body weight

**Figure 2:**
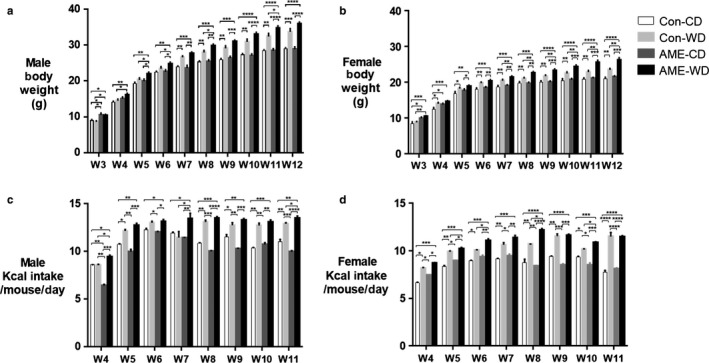
AME‐WD increased body wrights and calorie consumption. Data are presented as mean ± *SEM*. (a) Male body weights. (b) Female body weights. (c) Calorie intake per mouse per day in males. (d) Calories intake per mouse per day in females. *p < .05, **p < .01, ***p < .001, ****p < .0001, *n* = 20

**Figure 3:**
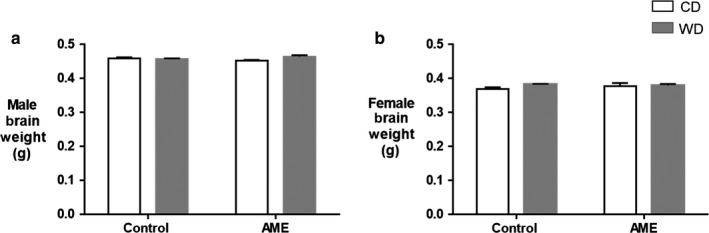
AME‐WD did not affect brain weights. Data are presented as mean ± *SEM*. (a) Brain weights in males. (b) Brain weights in females. *n* = 20

We investigated the effect of an adverse maternal environment combined with postweaning western diet (AME‐WD) on offspring body weight and brain weight. AME‐WD significantly increased body weight from postnatal week 3 to adulthood in line with increased calorie consumption (Figure 2). Similar results were also observed in Con‐WD group, indicating a diet effect. However, Con‐WD increased BW to a less extent compared to AME‐WD. At postnatal week 12, the end of experiment, AME‐WD offspring was significantly heavier compared to the control (Con‐CD) or postweaning WD (Con‐WD) or adverse maternal environment alone (AME‐CD) in both males (interaction: *F* = 1.892, *p* = .1745) and females (interaction: *F* = 5.566, *p* = .0223). Importantly, these data indicate an additive effect of AME and postweaning WD on body weight in females but not in males. However, AME with or without postnatal WD did not affect brain weight at the end of experiment (Figure 3). No sex differences in brain weight were observed.

### 3.2 AME‐WD decreased learning and memory in adult males

**Figure 4:**
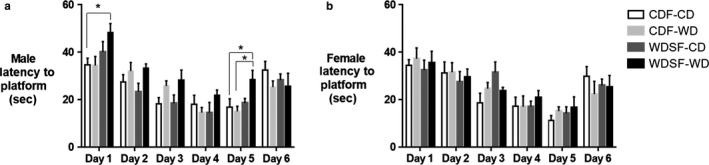
AME‐WD decreased learning and memory assessed by Morris Water Maze (MWM) test in male mice. Data are presented as mean ± *SEM*. (a) Latency to platform in males. (b) Latency to platform in females. No differences were found among female groups. *p < .05, *n* = 8 litters/group

Learning and memory was assessed in male and female adult mice using the Morris Water Maze (MWM) test. During testing mice are required to find the platform, and increased time finding the platform indicates an impairment in learning and memory of nonspatial (visible platform on testing day 1) or spatial (hidden platform on testing days 2–5) information (Bromley‐Brits, Deng, & Song, 2011). In this test, male AME‐WD took significantly more time finding the platform compared to male control mice (Con‐CD) on days 1 and 5 of testing, however, no additive effects were noted (day 1 interaction: *F* = 0.7338, *p* = .3992; day 5 interaction: *F* = 2.552, *p* = .1218) (Figure 4a). In addition, male AME‐WD took significantly more time finding platform on test day 5 compared to male Con‐WD (interaction: *F* = 2.552, *p* = .1218) (Figure 4a). No differences were found in female mice among groups in time finding platform (Figure 4b). No differences were found in swimming motivation, ability or speed among the four group mice in either sex (data not shown). Together, these data suggest that AME‐WD significantly impaired both nonspatial and spatial learning and memory in males relative to controls (Con‐CD) and impaired spatial learning and memory relative to postweaning WD mice (Con‐WD) in males.

Anxiety‐like behaviors were assessed in adult mice with the open field test (OFT) and elevated plus maze (EPM) test. AME‐WD females traveled significantly more distance than CD‐CD females in the OFT, however, no other differences were detected in either sex or group (Table 2 & Figures S1–S2).

**Table 2:** AME‐WD did not affect anxiety‐like and depression‐like behaviors in the open field (OF), the elevated plus maze (EPM), and the forced swim (FS) tests

Test	Measure	Sex	Con‐CD	Con‐WD	AME‐CD	AME‐WD
**OF**	Mean speed (m/s)	M	0.055 ± 0.002	0.051 ± 0.004	0.053 ± 0.003	0.047 ± 0.002
		F	0.054 ± 0.003	0.056 ± 0.003	0.056 ± 0.005	0.054 ± 0.004
	Total distance traveled (m)	M	98.2 ± 4.2	91.3 ± 6.5	94.8 ± 5.2	89.0 ± 4.6
		F	97.6 ± 5.5	101.8 ± 5.2	101.1 ± 9.7	96.9 ± 6.9
	Center zone entries (t)	M	289 ± 14.7	305 ± 10.3	293.5 ± 9.5	300.0 ± 15.6
		F	302.4 ± 23.4	292.7 ± 10.3	295.0 ± 20.7	283.7 ± 16.8
	Distance traveled in center zone (m)	M	59.9 ± 2.7	56.8 ± 4.2	56.1 ± 4.1	55.7 ± 3.5
		F	53.3 ± 5.2	56.1 ± 3.9	52.9 ± 4.9	57.1 ± 6.0
**EPM**	Open arm entries (%)	M	33.0 ± 1.4	35.7 ± 2.3	34.5 ± 0.9	36.4 ± 0.9
		F	31.5 ± 1.7	30.8 ± 3.0	33.2 ± 2.2	29.5 ± 3.1
	Time in open arm (%)	M	29.5 ± 3.0	28.2 ± 4.9	29.8 ± 2.4	36.5 ± 2.9
		F	12.9 ± 1.1	9.2 ± 3.1	13.0 ± 2.8	22.9 ± 3.3
	Total distance traveled(m)	M	1.0 ± 0.2	1.1 ± 0.3	1.3 ± 0.3	1.4 ± 0.2
		F	1.6 ± 0.2	0.8 ± 0.4	1.5 ± 0.4	2.7 ± 0.5^#^
	Center entries (*n*)	M	24.4 ± 2.0	22.6 ± 3.1	28.1 ± 1.6	31.4 ± 2.4
		F	15.1 ± 2.0	10.1 ± 2.1	17.5 ± 4.2	21 ± 2.7
**FS**	Immobility time (s)	M	188.8 ± 7.3	191.5 ± 9.5	189.5 ± 8.4	197.1 ± 8.2
		F	168.0 ± 8.1	165.6 ± 12.0	159.9 ± 6.6	168.9 ± 9.3

Note

Data are shown as mean ± *SEM*, for Con‐CD, Con‐WD, AME‐CD, and AME‐WD.

Abbreviations: female: F, meters: m; male: M; meter/second: m/s; numbers: n; percent: %; second: s; times: t..

^#^ *p* < .05 when AME‐WD compared to Con‐WD. *N* = 8 litters/group.

Depression‐like behaviors were assessed in adult mice using immobility time in a forced swim test (FST). No differences were observed in FST among four groups in either sex (Table 2 & Figure S3).

**Figure 5:**
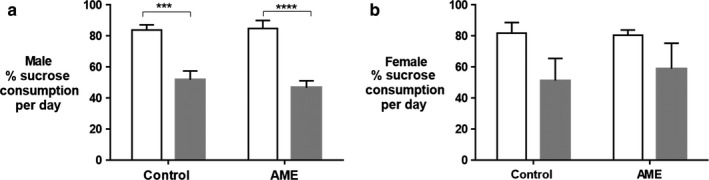
Western diet (WD) decreased sucrose consumption in adult male mice. Data were presented as mean ± *SEM*. (a) Percent of sucrose consumption per day in males. (b) pPercent of sucrose consumption per day in females. No differences were found among female groups. ***p<.001, ****p<.0001, *n* = 8 litters/group

Depression‐like behaviors were also assessed in adult mice using sucrose preference test (SPT). In SPT, sucrose consumption is used as a measure of anhedonia, a core feature of depression in humans. Male AME‐WD mice consumed significantly less sucrose than male control mice (Con‐CD). Additionally, male AME‐WD mice consumed less sucrose than male mice exposed to AME only (AME‐CD). However, AME‐WD did not differ from Con‐WD and additionally, AME‐CD did not differ from Con‐CD (interaction: *F* = 0.4455, *p* = .5101), we suspect diet may affect this behavior (Figure 5a). There were no significant differences in the female mice (Figure 5b).

### 3.3 AME‐WD increased hippocampal GR expression in males

**Figure 6:**
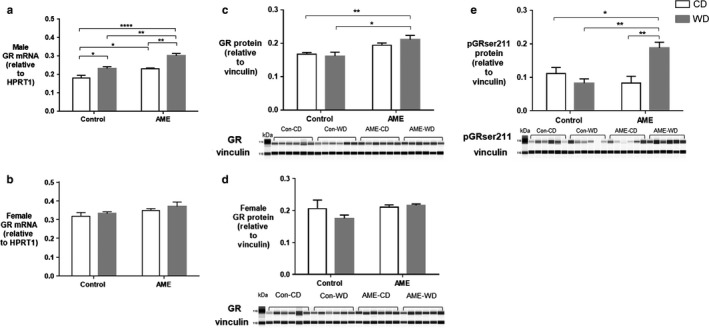
AME‐WD increased total GR expression in male hippocampus. Data are presented as mean ± *SEM*. (a) Total GR mRNA levels in males. (b) Total GR mRNA levels in females. (c) Total GR protein levels in males. (d) Total GR protein levels in females. (e) pGRser211 protein levels in males, with an additive effect of AME and WD. *p < .05, **p < .01, ****p < .0001, *n* = 6 litters/group

We next investigated the effect of AME‐WD on hippocampal GR expression and serum corticosterone levels. AME‐WD significantly increased hippocampal total GR mRNA levels in male (interaction: *F* = 0.6589, *p* = .427) (Figure 6a) but not in female mice (Figure 6b) when compared to either Con‐CD or Con‐WD or AME‐CD. Similarly, AME‐WD also significantly increased total GR protein levels in males (interaction: *F* = 1.317, *p* = .2662) (Figure 6c) but not in females (Figure 6d) when compared to either Con‐CD or Con‐WD, although no additive effects were noted. Additionally, protein levels of phospho‐GR Ser 211, the active form of GR (Figure 6e) were increased in AME‐WD compared to either Con‐CD, Con‐WD, or AME‐CD in male mice with an additive effect of AME and WD (interaction: *F* = 12.93, *p* = .0021). A single hit of either postweaning WD or AME (Con‐WD or AME‐CD) also increased GR mRNA but did not affect protein concentration of pGRSer211 (Figure 6a&e). Together, the combination of the prenatal AME and postweaning WD insults was greater than either individual insult on hippocampal GR expression. Notably, there were not significant differences among four groups in serum corticosterone levels in either sex (Table 3 & Figure S4). Finally, since only male mice exhibited behavioral and GR expression changes, we focused on male mice for subsequent molecular studies.

**Table 3:** AME‐WD did not affect serum corticosterone levels (pg/ml)

Sex	Con‐CD	Con‐WD	AME‐CD	AME‐WD
M	883.6 ± 158.4	1,047.5 ± 169.5	1,332.7 ± 316.7	1,242.8 ± 103.2
F	1,073.6 ± 226.9	3,030.5 ± 646.1	2,014.7 ± 642.7	1,229.8 ± 193.5

Note

Data are shown as mean ± *SEM*, for Con‐CD, Con‐WD, AME‐CD, and AME‐WD. Abbreviations: M: male; F: female. *N* = 8 litters/group

### 3.4 AME‐WD increased hippocampal GR 1.7 mRNA levels in males

**Figure 7:**
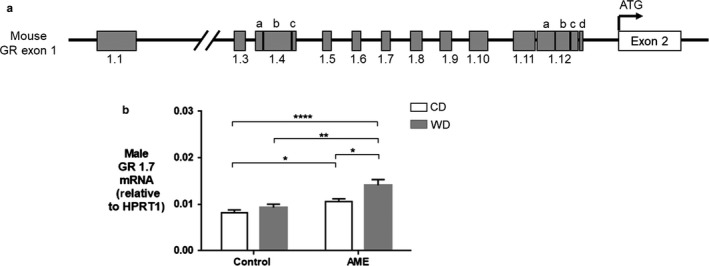
AME‐WD increased GR 1.7 mRNA levels in male hippocampus. (A) Schematic representation of mouse exon 1 of GR gene. Alternative splicing of exon 1 generates multiple mRNA. Mouse exon 1.4 has three subvariants (a, b, c) and exon 1.12 has four subvariants (a, b, c, d) (Bockmuhl et al., 2011). (B) GR 1.7 mRNA levels in males. Data are presented as mean ± *SEM*. *p < .05, **p < .01, ***p < .001, *n* = 6 litters/group

Hippocampal GR1.7 demonstrated dynamic change in expression in response to AME and WD. Hippocampal GR 1.7 mRNA levels were significantly increased in AME‐WD compared to all other groups in males (Figure 7), however, no additive effects of AME and WD were noted (interaction: *F* = 2.18, *p* = .1562). Hippocampal GR 1.7 mRNA levels were also significantly increased in AME‐CD compared to Con‐CD.

### 3.5 AME‐WD decreased hippocampal CpG methylation at exon 1.7 promoters in males

**Figure 8:**
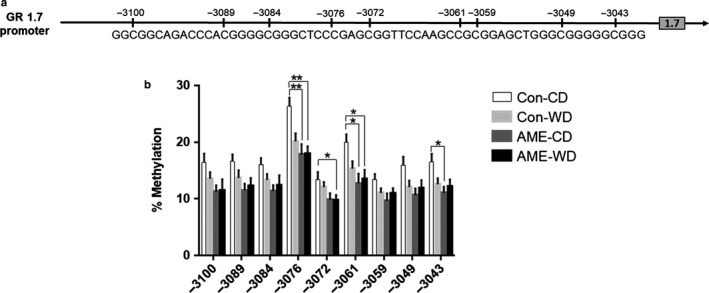
AME‐WD and AME alone decreased CpG methylation at GR 1.7 promoter in male hippocampus. (a) Schematic representation of GR 1.7 promoter and the sequence for CpG methylation analysis. Vertical lines indicate the location of the CpG sites examined relative to translation start site set as + 1 in exon 2. (b) Percent of methylation at 9 CpG sites examined. Data are presented as mean ± *SEM*. *p < .05, **p < .01, *n* = 6 litters/group

Given the consistent changes in GR 1.7 mRNA and GR protein expression in males, we next investigated DNA methylation at the GR 1.7 promoter as a potential contributing mechanism. Nine CpG sites in the GR 1.7 promoter region were examined (Figure 8a). Compared to control (Con‐CD), CpG methylation trended to decrease at all sites examined in the 1.7 promoter in AME‐WD male hippocampi with statistical significance at sites −3076 (interaction: *F* = 4.602, *p* = .0476), −3072 (interaction: *F* = 0.2508, *p* = .6238), and −3061 (interaction: *F* = 2.922, *p* = .1080). CpG methylation also was significantly decreased at sites −3076, −3061, and −3043 (interaction: *F* = 4.047, *p* = .0626) in AME‐CD mice (Figure 8b).

### 3.6 AME‐WD increased hippocampal expression of FKBP4 and FKBP5 in males

**Figure 9:**
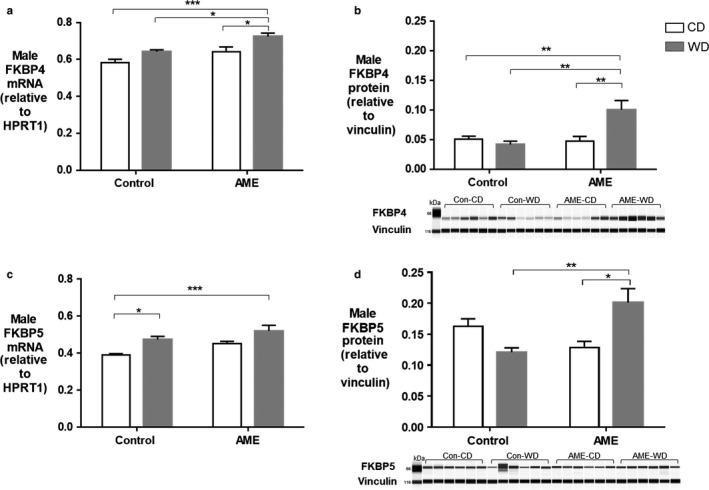
Expression of GR co‐chaperon protein FKBP4 and FKBP5 in male hippocampus. Data are presented as mean ± *SEM*. (a) FKBP4 mRNA levels. (b) FKBP4 protein levels, an additive effect of AME and WD was noted. (c) FKBP5 mRNA levels. (d) FKBP5 protein levels, an additive effect of AME and WD was noted. *p < .05, **p < .01, ***p < .001, *n* = 6 litters/group

FKBP4 and FKBP5 are important regulators of GR signaling. We investigated the effect of AME‐WD on hippocampal expression of FKBP4 and FKBP5. AME‐WD significantly increased hippocampal mRNA levels of FKBP4 (interaction: *F* = 0.3495, *p* = .5610) (Figure 9a) compared to all other groups in males. Interestingly, there was an additive effect of AME and WD on FKBP4 protein levels (interaction: *F* = 10.69, *p* = .0038) (Figure 9b).

AME‐WD also significantly increased hippocampal expression of FKBP5 compared to control (Con‐CD) (interaction: *F* = 0.1474, *p* = .7051) (Figure 9c) although protein content was not significantly changed (Figure 9d). However, FKBP5 protein content was significantly increased in AME‐WD compared to AME alone (AME‐CD) and western diet alone (Con‐WD) (Figure 9d) with an additive effect of AME and postweaning WD (interaction: *F* = 15.57, *p* = .0013).

## 4 DISCUSSION

The central findings of this study are that AME and WD together decrease learning and memory in male adult offspring. This impaired learning and memory was concurrent with the changes in hippocampal GR1.7 expression, DNA methylation, and GR signaling relative to AME or postweaning WD alone. This combination is relevant considering the previous literature associating neurocognitive impairment and hippocampal GR biology.

Using our novel model of chronic adversity including an AME and postweaning WD, we found that both insults were necessary to cause decreases in learning and memory. These finding are in accordance with a two‐hit model and suggest that both AME and WD are necessary for impaired cognition in adulthood. Observational human studies parallel these findings as demonstrated by Nomura et al. (2012) who found maternal gestational diabetes mellitus and low socioeconomic status, especially in combination increase the risk for lower IQ, poorer language, temperament behavioral functioning, attention deficits, etc. later in life. Pervious animal models by others have not recapitulated these human findings entirely (Tozuka et al., 2010; Zieba, Uddin, Youngson, Karl, &amp; Morris, 2019) or found that the combination of two environmental insults rescued memory impairment (Arcego et al., 2016). Importantly, these other animal models employed different types and timing of environmental stressors on the offspring. It is well known that animal models are keenly sensitive to stress and these differences are likely to drive diverse outcomes (Bogdanova, Kanekar, D'Anci, & Renshaw, 2013).

Our findings of reduced sucrose consumption significantly in the two bottles choice paradigm in both Con‐WD and AME‐WD male mice and trending to decrease in females suggest that WD influences this phenotype. Given that our WD diet consists of high sucrose, we speculate that Con‐WD and AME‐WD mice may be satiated by the sucrose in the western diet and drink less in SP test in our model. Furthermore, we found that neither AME‐WD nor AME alone affect depressive‐like behavior measured by FST nor anxiety‐like behavior determined by OF test and EPM test. However, Maniam, et al. have demonstrated that male rats exposed to early life stress either induced by either maternal separation (Maniam & Morris, 2010) or simulated by limited nesting material postnatally (Maniam, Antoniadis, Le, Morris, 2016) display anxiety‐like and depression‐like behaviors without cognitive impairments. While postweaning high‐fat diet reverses depressive‐like and anxiety‐like phenotypes. The type of stress and timing of it is an important different between these studies and ours. Our model uses maternal prenatal stress which the offspring are not directly exposed to. This type of stress may not be the right timing or strength to effect anxiety and depressive behaviors.

Interestingly, the behavior differences described here were only exhibited by male mice, which also mirrors some human (Alastalo et al., 2013; de Kloet et al., 2005) and animal studies (Arp et al., 2016; Gelineau et al., 2017; Maniam et al., 2010; Naninck et al., 2015). Alastalo et al. (2013) have reported that temporarily childhood parental separation during World War II increased the risk for impaired physical and psychosocial functioning in adult men but not in adult women. Naninck et al. (2015) showed that limited nesting and bedding material from postnatal day 2–9 impaired cognitive functions in hippocampus‐dependent tasks more prominently in male adult mice. Furthermore, high‐fat diet exposure decreases activity in open field test in adult male but not in female mice (Gelineau et al., 2017). Similarly, in current study, we found that both the effects of AME‐WD on learning and memory and the effects of postweaning WD on depression‐like behavior were only apparent in males. These observations support the notion that males have higher sensitivity to early life stress and more vulnerable to develop cognitive deficits (Arp et al., 2016; Naninck et al., 2015). Unfortunately, the underlying mechanism for this sex‐dependent early life environment sensitivity is not fully understood. We speculate that sex hormones may play an important role in this sex difference.

Importantly, AME‐WD induced sex specific behavior changes also resulted in sex specific hippocampal GR changes.

Our finding of AME‐WD induced cognitive impairment was concurrent with increased hippocampal GR expression in male mice, suggests hippocampal GR plays a role in this impaired neurobehavioral phenotype. While previous literature supports this association (Arp et al., 2016; Revsin et al., 2009), we have also examined important changes in GR variants and regulation. Transcriptional regulation of GR gene relies in part upon the regulation of multiple exon 1 mRNA variants (Bockmuhl et al., 2011; Jacobson, 2014; Turner et al., 2005). GR 1.7 expression has been shown to be consistent with total GR expression in the hippocampus and particularly vulnerable to early life events (Ke et al., 2010; McGowan et al., 2009; Weaver et al., 2004). However, these studies focused on the effect of a single environmental factor on GR exon 1 variant expression. In the present study, we found that the combination of two environmental challenges, AME and WD significantly increased GR and GR 1.7 mRNA levels to a greater extent in male hippocampi although other single hit groups (Con‐WD and AME‐CD) also increased total GR and GR 1.7 expression when compared to control (Con‐CD) mice. We speculate that increased GR1.7 expression may contribute to upregulation of total GR expression in male AME‐WD mouse hippocampi.

Interestingly, we also found that either AME‐WD or AME alone decreased DNA methylation at limited CpG sites in GR 1.7 promoter compared to controls, suggesting a prenatal effect. While AME alone did not significantly increased GR 1.7mRNA levels, when performing multiple group comparisons, AME alone did significantly increase GR 1.7 mRNA levels compared to controls. Our findings are consistent with what have previously reported that DNA methylation at the GR 1.7 promoter has been shown to regulate GR 1.7 expression in the hippocampus of humans (McGowan et al., 2009) and rodents (Weaver et al., 2004, 2007) exposed to early life stress. Given that postnatal WD did not affect DNA methylation at GR 1.7 promoter, we speculate that DNA methylation at the promoter is not the only mechanism that affects GR 1.7 mRNA levels in AME‐WD mice. Other mechanisms including, histone modifications (Ke et al., 2010) and transcription factor binding, may also be involved in the upregulation of GR 1.7 expression in AME‐WD male mouse hippocampi.

Increased pGRser211 (corresponding to Ser232 of mouse GR) protein levels in AME‐WD suggest that AME‐WD may increase GR transcriptional activity (Chen et al., 2008; Wang et al., 2002). Consistent with this, we also found AME‐WD increased FKBP4 as well as FKBP5, a target and regulator of GR. This additive effect of AME and postweaning WD indicate disruption of GR, FKBP4 and FKBP5 interactions in AME‐WD male mouse hippocampus. Our results and others (Brkic, Petrovic, Franic, Mitic, & Adzic, 2016) support the notion that phosphorylation of GR could be a potential biomarker of neuropsychiatric vulnerability in healthy adults (Simic et al., 2013).

All molecular analysis performed in this study utilized whole homogenized hippocampus, thus limiting our ability to understand the contribution of hippocampal subfields but allowed for a comprehensive hippocampal investigation. In doing so, we have identified that adverse maternal environment and postweaning western diet impair learning and memory concurrently with increased expression of total GR and GR 1.7 expression as well as decreased DNA methylation at GR1.7 promoter in male mouse hippocampus. We speculate that decreased DNA methylation at the GR 1.7 promoter may play a role in the upregulation of GR 1.7 expression that may contribute to GR upregulation in the hippocampus. Upregulated hippocampal GR may subsequently contribute to cognition impairment in AME‐WD male mice.

## CONFLICT OF INTEREST

No conflict of interest, financial or otherwise, are declared by the authors.

## Supporting information



Fig S1

Fig S2

Fig S3

Fig S4
