# Supplementary figures and images for: Adverse maternal environment and western diet impairs cognitive function and alters hippocampal glucocorticoid receptor promoter methylation in male mice
Source: Physiol Rep. 2020 Apr 25;8(8):e14407. doi: 10.14814/phy2.14407 (PMC7183239; doi:10.14814/phy2.14407)

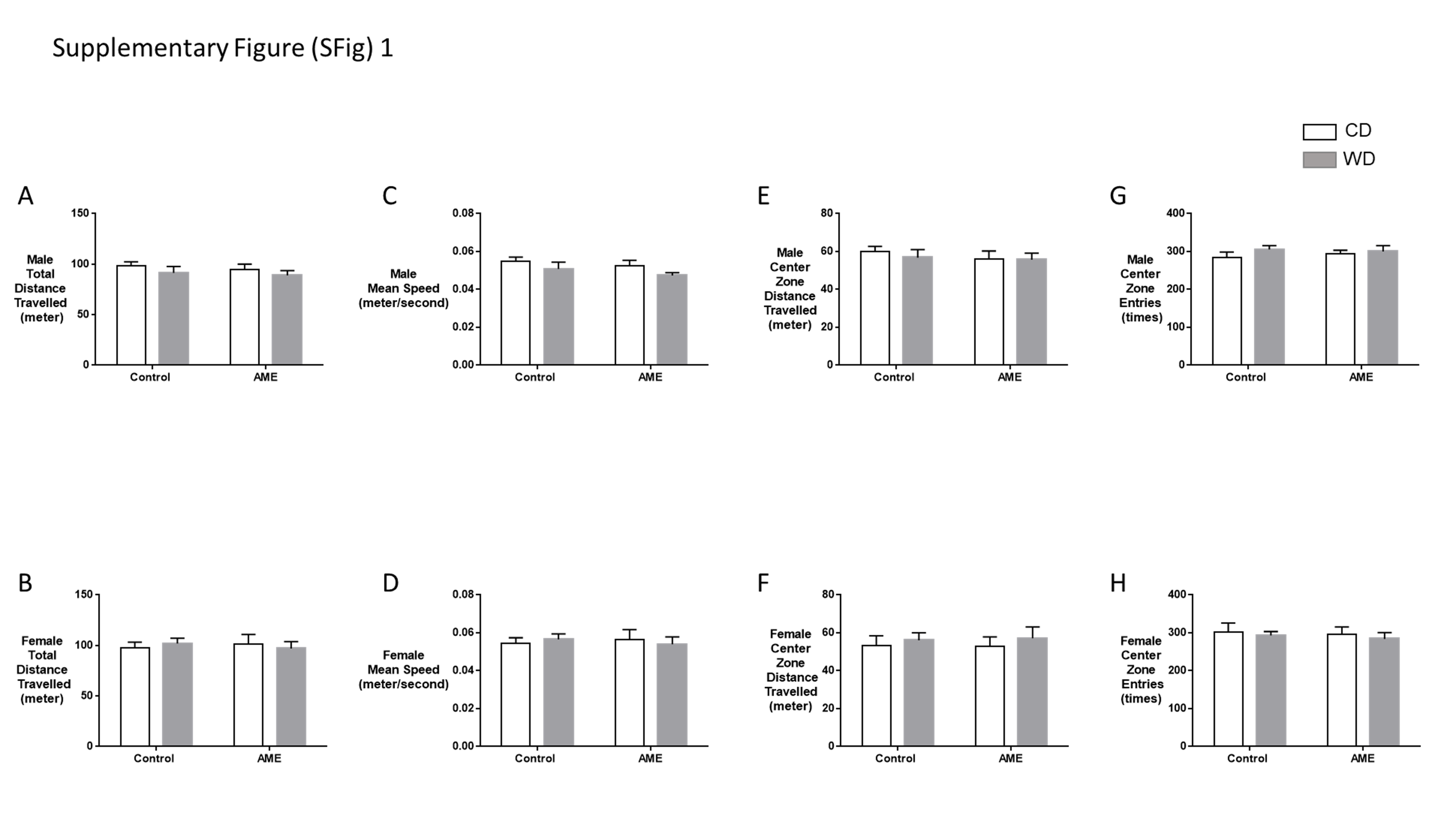

Supplement: Supplementary file 1 — Fig S1 [file PHY2-8-e14407-s001.tif]

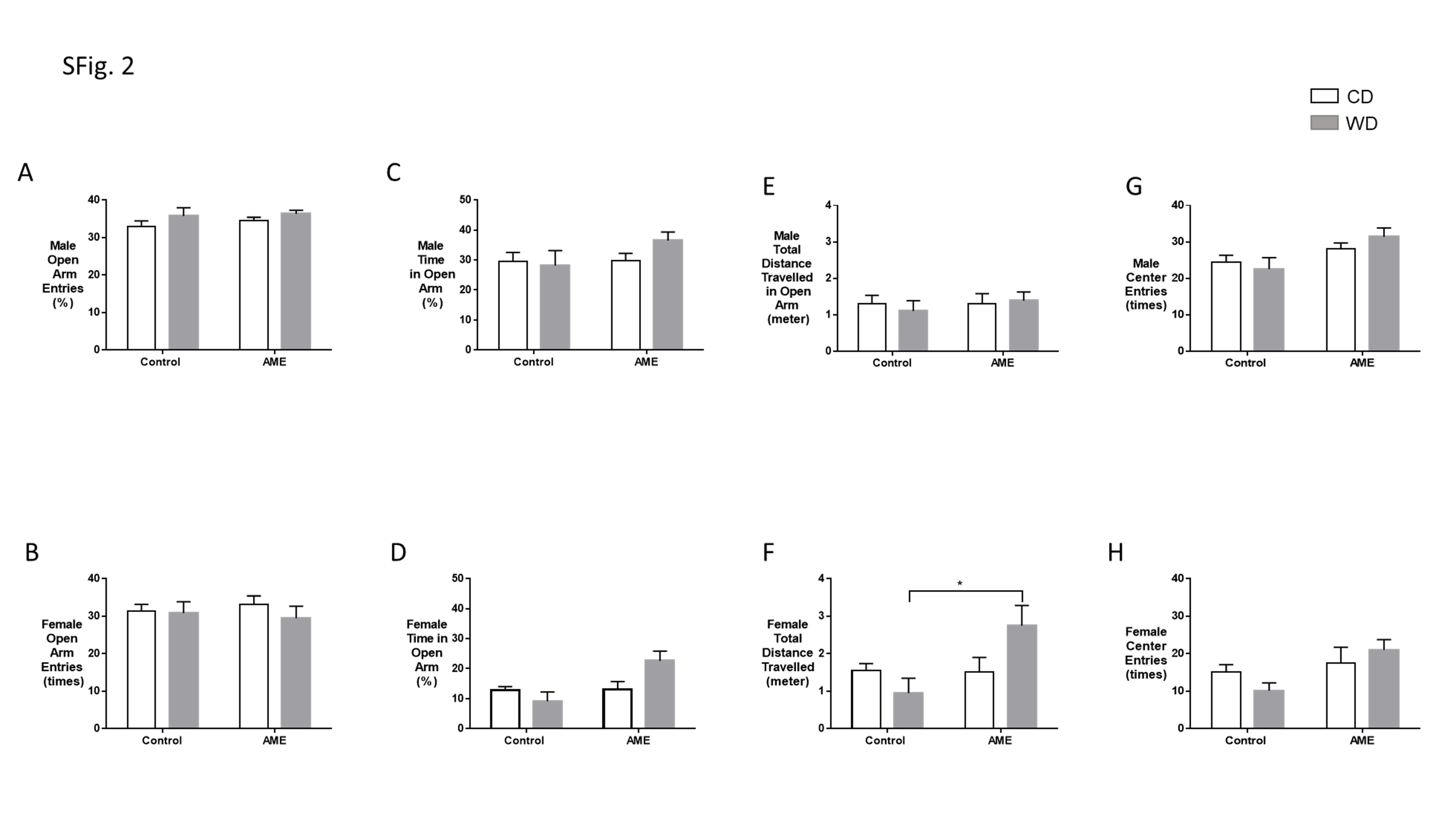

Supplement: Supplementary file 2 — Fig S2 [file PHY2-8-e14407-s002.tif]

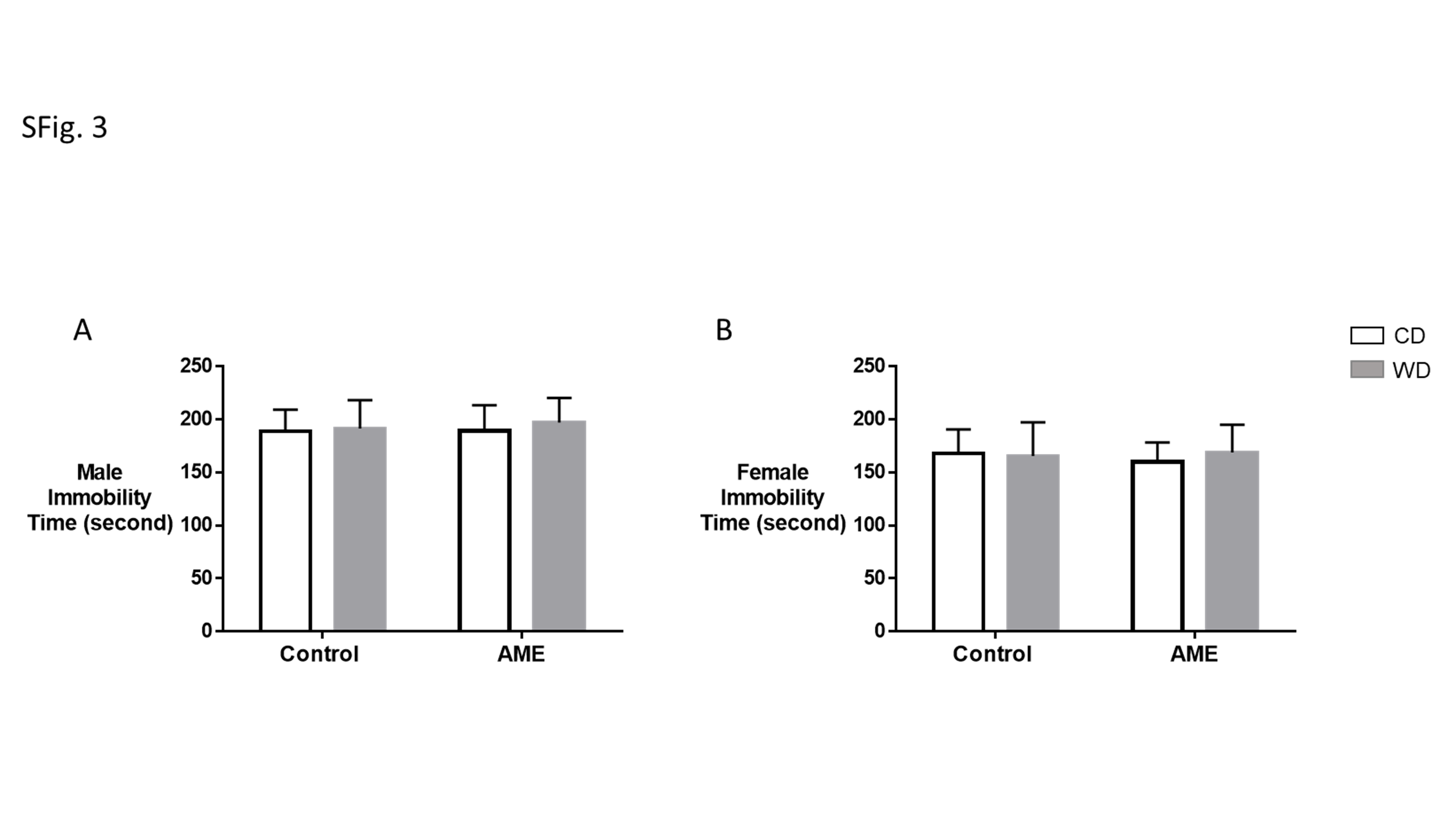

Supplement: Supplementary file 3 — Fig S3 [file PHY2-8-e14407-s003.tif]

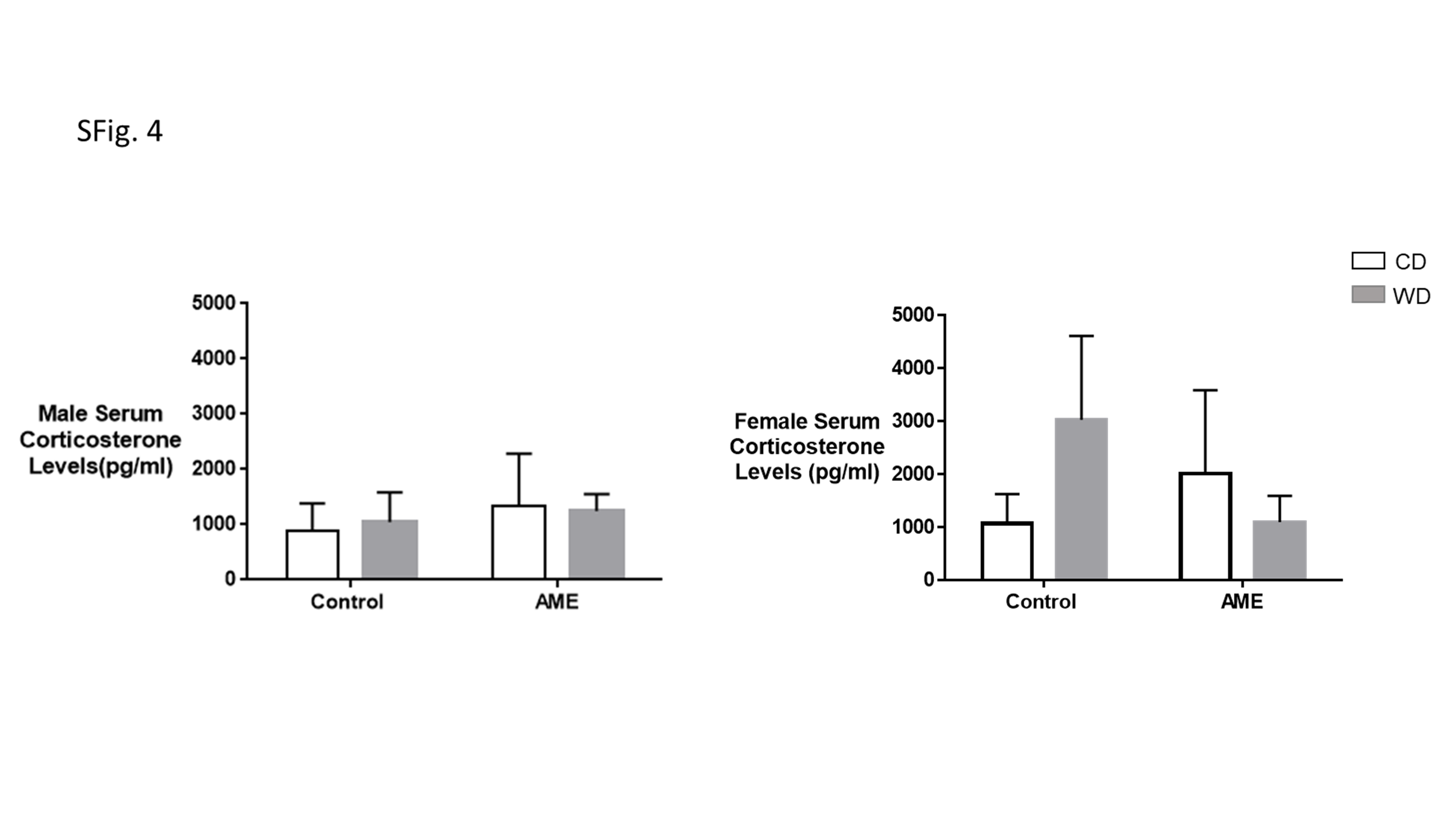

Supplement: Supplementary file 4 — Fig S4 [file PHY2-8-e14407-s004.tif]
